# Supplementary material for: Exploring Evolutionary Pathways and Abiotic Stress Responses through Genome-Wide Identification and Analysis of the Alternative Oxidase (AOX) Gene Family in Common Oat (Avena sativa)
Source: Int J Mol Sci. 2024 Aug 29;25(17):9383. doi: 10.3390/ijms25179383 (PMC11395127; doi:10.3390/ijms25179383)
Supplement: Supplementary file 1 [file ijms-25-09383-s001.zip › Figure S1.pdf]

>AsAOX1
MSSRMAGSLLLRHAGGASRRLFSATATSPAARPLLAGGEGVQGSWVRLMSTSAGTQAKDE
AAKAAAAAAASNKGDGEKKEVAVNSYWGIEQSQKLVREDGTEWKWSCFRPWETYSADTTI
DLTKHHVPKTMLDKIAYWTVKSLRFPTDIFFQRRYGCRAMMLETVAAVPGMVGGMLLHLR
SLRRFEQSGGWIRALLEEAENERMHLMTFMEVAQPRWYERALVITVQGVFFNAYFFGYLI
SPKFAHRVVGYLEEEAVHSYTEFLKDLEDGKIDNVPAPAIAIDYWRLPANATLKDVVTVV
RADEAHHRDVNHFASDVYYQGMQLKATPAPIGYH*
>AsAOX2
MSKRIMDATFRETTAPAEPAPRRSRFAVPPIRTSQHETTSFHQSPQQHNHRSVPLNFDLP
SHTPHKRLLILVKFDHAMSSRMAGATLLRHLGPRLFAAAEPASGGLAASARGIMPAAARI
LPARMASTDAKQVAAEKPEEAATTEQSSTKNAVASYWGVQPRKLVREDGTDWPWFCFTPW
DTYRADTSIDVKKHHEPRTLPDKVAYYAVRSLRVPMDLFFQRRHSSHALLLETVAAVPPM
VGGVLLHLRSLRRFEHSGGWIRALMEEAENERMHLMTFLEVTQPRWWERALVVAAQGVFF
NAYFVGYLVSPKFAHRFVGYLEEEAVHSYTEYLKDLEAGIIENTPAPAIAIDYWRLPADA
TLKDVVTVVRADEAHHRDANHYASVRHPLPGTEAQGGACADRVPLINSNFNCQIDR*
>AsAOX3
MSSRMAGSLLLRHAGGASRRLFSATATSPAARPLLAGGEGVQGSWVRLMSTSAGTQAKDE
AAKAAAAATAANKGDGEKKEVAVNSYWGIQQSQKLVREDGTEWKWSCFKPWETYSADTSI
DLTKHHVPKTMLDKIAYWTVKSLRFPTDIFFQRRYGCRAMMLETVAAVPGMVGGMLLHLR
SLRRFEQSGGWIRALLEEAENERMHLMTFMEVAQPRWYERALVITVQGVFFNAYFFGYLI
SPKFAHRVVGYLEEEAVHSYTEFLKDLEDGKIDNVPAPAIAIDYWRLPANATLKDVVTVV
RADEAHHRDVNHFASDVYYQGMQLKATPAPIGYH*
>AsAOX4
MSSRMAGATLLRHLGPRLFAAAEPASGGLAASARGIMPAAARIFPARMASTDAKQVATEK
PEEAATTEQSNIKNAVASYWGVQPRKLVREDGTEWPWFCFTPWDTYRADTSIDVKKHHEP
RTLPDKVAYYTVRSLRVPMDLFFQRRHSSHALLLETVAAVPPMVGGVLLHLRSLRRFEHS
GGWIRALMEEAENERMHLMTFLEVTQPRWWERALVVAAQGVFFNAYFVGYLVSPKFAHRF
VGYLEEEAVHSYTEYLKDLEAGIIENTPAPAIAIDYWRLPADATLKDVVTVVRADEAHHR
DANHYASDIHYQGMKLKEAPAPIGFH*
>AsAOX5
MSSRMAGATLLRHLGPRLFAAAAEPASGGLAASARGIMLPAAARIFPARMASTDAKQVAA
EKPEEAAATEQSDTKNAVASYWGVQPRKLVREDGTEWPWFCFTPWDTYRADTSIDVKKHH
EPRTLPDKVAYYAVRSLRVPMDLFFQRRHSSHALLLETVAAVPPMVGGVLLHLRSLRRFE
HSGGWIRALMEEAENERMHLMTFLEVTQPRWWERALVVAAQGVFFNAYFVGYLVSPKFAH
RFVGYLEEEAVHSYTEYLKDLKAGIIENTPAPAIAIDYWRLPADATLKDVVTVVRADEAH
HRDANHYASDIHYQGLKLKEAPAPIGFH*
>AsAOX6
MSSRMAGSLLLRHAGGASRRLFSATATSPAARPLLAGGEGVQGSWVRLMSTSAGTQAKDE
AAKAAAAAAAANKGDGEKKEVAVNSYWGIEQSQKLVREDGTEWKWSCFRPWETYSADTTI
DLTKHHVPKTMLDKIAYWTVKSLRFPTDIFFQRRYGCRAMMLETVAAVPGMVGGMLLHLR
SLRRFEQSGGWIRALLEEAENERMHLMTFMEVAQPRWYERALVITVQGVFFNAYFFGYLI
SPKFAHRVVGYLEEEAVHSYTEFLKDLEDGKIDNVPAPAIAIDYWRLPANATLKDVVTVV
RADEAHHRDVNHFASDVYYQGMQLKATPAPIGYH*
>AsAOX7
MAGVASASAAPLPAAPSSSSSSPAGRSPPSLLPLRARRLHGPAVLATGMGRRFRAEAIRT
QREKAEQEIEVSPVEESFPVRETGAPEAAAANDPPAPTEDDDWVVRFEQSFNIFLTDSII
VILDALYRDRDYARFFVLETIARVPYFAFISVLHLYETFGWWRRADYIKVHFAESMNEFH
HLLIMEELGGNSVWVDRFLARFSAFFYYFMTVAMYMLSPRMAYHFSECVERHAYSTYDKF
LKLNGEELKKLPAPEAAINYYMNEDLYLFDEFQTSRAPCSRRPKVDNLYDVFVNIRDDEA
EHCKTMKACQTHGNLRSPHSMPSNIETDEECIVPGNDCEGIMDCVKKSLTSED*
>AsAOX8
MSSWSVLARRHVVPSPSQKLARLQVRETAATSSASRVGHRFAGSSSSAMRSRVAGSVLLR
HLGPRVFGPATPASGSRPLLVGGEGGVWARLLATSAAEAAKEEVAASKDNVASTAAAKAE
AVQAAKEGEKGAVVSSYWGIVPAKLVNKDGAEWKWSCFRPWEAYTSDTSIDLTKHHQPKI
LLDKIAYWTVKSLRVPTDIFFQRRYGCRAMMLETVAAVPGMVGGMLLHLRSLRRFEHSGG
WIRALLEEAENERMHLMTFMEVADPKWYERALVLAVQGVFFNAYFVGYLLSPKFAHRVVG
YLEEEAIHSYTEFLRDLEAGKIENVPAPRIAIDYWRLPADATLKDVVTVVRADEAHHRDV
NHFAADIHFQGLELNKTPAPLGYH*
>AsAOX9
MAGVASASAAPLPAAPSSSPAARSPPSFLPLRARRLHGPAVLATGRGRTFRAEAIKTQRE
KAEQEAEVSAVEESFPVREEAGPPEAAAADDPPSPTDDDDDWVVRFEQQFNIFLTDSIIV
ILDALYRDRDYARFFVLETIARVPYFAFISVLHLYETFGWWRRADYIKVHFAESMNEFHH
LLIMEELGGNSVWVDRFLARFSAFFYYFMTVAMYMLSPRMAYHFSECVERHAYSTYDKFL
KLNGDELKKLPAPEAAVNYYMNEDLYLFDEFQTSRVPCSRRPKIDNLYDVFVNIRDDEAE
HCKTMKACQTHGNLRSPHSMPSNIETDTECAVPENDCEGIMDCVKKSLSSED*
>AsAOX10
MAGLASASASPVPAALPSNFAGFSPRSFLPSPPRARRPVVLVSRRRGRFRAEATQREKTR
QETAVEESFPVRDAAGPPEAVALTDDDDWVVRLEQSFNIFLTDFIIAILDLLYRDRDYAR
FFVLETIARVPYFAFISVLHLYETFGWSRRADYIKVHFAESMNEFHHLLIMEELGGNSEW
IDRFLARFSAFFYYFIAVGMYMLSPRMAYHFSECVERHAYSTYDKFLKVNGEELIKLPAP
EAAINYYMNEDLYLFDEFQTSRAPCSRRPKVENLYDVFVNIRDDEAEHCKTMAACQTPGN
LRSPHSMDDSVKTSIANDD*
>As6CLAOX11

MSTWRALARRHVVPSPSQKLARLQVREPSATSFASRAGHQLAGSSSSAMRSRVAGSVLLR
HLGPRVFGSATPAAASRPLLVGGEGGGVWARLLATSAAEAAKEEVAASKDNVASTAAAKA
EAVQAAKEGEKGAVVSSYWGIVPAKLVNKDGAEWKWSCFRPWEAYTSDTSIDLTRHHQPK
ILLDKIAYWTVKSLRVPTDIFFQRRYGCRAMMLETVAAVPGMVGGMLLHLRSLRRFEHSG
GWIRALLEEAENERMHLMTFMEVADPKWYERALVLAVQGVFFNAYFVGYLLSPKFAHRVV
GYLEEEAIHSYTEFLRDLEAGKIENVPAPRIAIDYWRLPADATLKDVVTVVRADEAHHRD
VNHFAADIHFQGLELNKTPAPLGYH*
>As6DLAOX12
MRSRVAGSVLLRHLGPRVFGPATPASGSRPLLVGGEGGMWARLLATSAAEAAKEEVAASK
DNVASTAAAKAEAVQAAKEGEKGAVVSSYWGIVPAKLVNKDGAEWKWSCFRPWEAYTSDT
SIDLTKHHQPKILLDKIAYWTVKSLRVPTDIFFQRRYGCRAMMLETVAAVPGMVGGMLLH
LRSLRRFEHSGGWIRALLEEAENERMHLMTFMEVADPKWYERALVLAVQGVFFNAYFVGY
LLSPKFAHRVVGYLEEEAIHSYTEFLRDLEAGKIENVPAPRIAIDYWRLPADATLKDVVT
VVRADEAHHRDVNHFAADIHFQGLELNKTPAPLGYH*
>OsAOX1
MALVQSARRAAGPAASRLFSTASVAAAGRSPVAGLPKALPRPAISSLWMVGAAAPPRRFA
GTAAVGGVDVTAPTATPPPAKKEESEKEAASYWGVAPTRLVKEDGTVWKWSCFRPWDTYE
ADVAIDLTKHHNPATLGDKVARWTVKSLRWPVDLFFQRRYGCRAMMLETVAAVPGMVAGA
VLHLRSLRRFEHSGGWIRALLEEAENERMHLMTFMEVSQPRWYERALVVAVQGAFFNAYL
ASYLLSPRFAHRIVGYLEEEAVHSYTEFLRDLDAGKIDDVPAPAIAIDYWRLPADATLRD
VVMVVRADEAHHRDVNHYASVRFPHSLTLCWTMHHVSQFRSKTKRRRNLNEFC
>OsAOX2
MGSRAAGSVLLRHLCPRVSSSTSAAAHAHAQRPPLAGAGGGGVALWARLLSTSAAAAKEE
TAASKENTGSTAAAKAEATKAAKEGPASATASPVASSYWGIEASKLASKDGVEWKWSCFR
PWETYSPDTTIDLKKHHEPKVLLDKVAYWTVKALRVPTDIFFQRRYGCRAMMLETVAAVP
GMVGGMLLHLRSLRRFEHSGGWIRALLEEAENERMHLMTFMEVAKPRWYERALVLAVQGV
FFNAYFLGYLLSPKLAHRVVGYLEEEAIHSYTEYLKDIEAGKIENVPAPPIAIDYWRLPA
GATLKDVVVVVRADEAHHRDVNHFASDVHFQGMDLKDIPAPLDYH
>OsAOX3
MSSRMAGSAILRHVGGVRLFTASATSPAAAAAAAARPFLAGGEAVPGVWGLRLMSTSSVA
STEAAAKAEAKKADAEKEVVVNSYWGIEQSKKLVREDGTEWKWSCFRPWETYTADTSIDL
TKHHVPKTLLDKIAYWTVKSLRFPTDIFFQRRYGCRAMMLETVAAVPGMVGGMLLHLRSL
RRFEQSGGWIRTLLEEAENERMHLMTFMEVANPKWYERALVITVQGVFFNAYFLGYLLSP
KFAHRVVGYLEEEAIHSYTEFLKDLEAGKIDNVPAPAIAIDYWRLPANATLKDVVTVVRA
DEAHHRDVNHFASDIHYQGMELKQTPAPIGYH
>OsAOX4
MSSRMAGATLLRHLGPRLFAAEPVYSGLAASARGVMPAAARIFPARMASTSSAGADVKEG
AAEKLPEPAATAAAAATDPQNKKAVVSYWGIQPPKLVKEDGTEWKWLSFRPWDTYTSDTS
IDVTKHHEPKGLPDKLAYWTVRSLAVPRDLFFQRRHASHALLLETVAGVPGMVGGMLLHL
RSLRRFEQSGGWIRALLEEAENERMHLMTFLEVMQPRWWERALVLAAQGVFFNAYFVGYL
VSPKFAHRFVGYLEEEAVSSYTEYLKDLEAGKIENTPAPAIAIDYWRLPADATLKDVVTV
IRADEAHHRDLNHFASDIQQQGMKLKDTPAPIGYH
>OsAOX5
MAAVASASPLPAAAAPSTRCSPPPAFLPLRAHRPRVGTVATRRVFRAEAMRTQREKEQTE
VAVEESFPFRETAPPDEPLVTAEESWVVKLEQSVNIFLTESVITILDGLYRDRNYARFFV
LETIARVPYFAFISVLHMYETFGWWRRADYIKVHFAESWNEFHHLLIMEELGGNSLWVDR
FLARFAAFFYYFMTVAMYMVSPRMAYHFSECVERHAYSTYDKFIKLHEDELKKLPAPEAA
LNYYLNEDLYLFDEFQTARVPCSRRPKIDNLYDVFVNIRDDEAEHCKTMKACQTHGNLRS
PHSMQKCLETDTECVIPEDDCEGIVDCVKKSLVSKE
>AtAOX1
MMITRGGAKAAKSLLVAAGPRLFSTVRTVSSHEALSASHILKPGVTSAWIWTRAPTIGGM
RFASTITLGEKTPMKEEDANQKKTENESTGGDAAGGNNKGDKGIASYWGVEPNKITKEDG
SEWKWNCFRPWETYKADITIDLKKHHVPTTFLDRIAYWTVKSLRWPTDLFFQRRYGCRAM
MLETVAAVPGMVGGMLLHCKSLRRFEQSGGWIKALLEEAENERMHLMTFMEVAKPKWYER
ALVITVQGVFFNAYFLGYLISPKFAHRMVGYLEEEAIHSYTEFLKELDKGNIENVPAPAI
AIDYWRLPADATLRDVVMVVRADEAHHRDVNHFASDIHYQGRELKEAPAPIGYH
>AtAOX2
MMMSRRYGAKLMETAVTHSHLLNPRVPLVTENIRVPAMGVVRVFSKMTFEKKKTTEEKGS
SGGKADQGNKGEQLIVSYWGVKPMKITKEDGTEWKWSCFRPWETYKSDLTIDLKKHHVPS
TLPDKLAYWTVKSLRWPTDLFFQRRYGCRAMMLETVAAVPGMVGGMLVHCKSLRRFEQSG
GWIKALLEEAENERMHLMTFMEVAKPNWYERALVIAVQGIFFNAYFLGYLISPKFAHRMV
GYLEEEAIHSYTEFLKELDNGNIENVPAPAIAIDYWRLEADATLRDVVMVVRADEAHHRD
VNHYASDIHYQGRELKEAPAPIGYH
>AtAOX3
MITTLLRRSLLDASKQATSINGILFHQLAPAKYFRVPAVGGLRDFSKMTFEKKKTSEEEE
GSGDGVKVNDQGNKGEQLIVSYWGVKPMKITKEDGTEWKWSCFRPWETYKADLTIDLKKH
HVPSTLPDKIAYWMVKSLRWPTDLFFQRRYGCRAIMLETVAAVPGMVGGMLMHFKSLRRF
EQSGGWIKALLEEAENERMHLMTFMEVAKPKWYERALVISVQGVFFNAYLIGYIISPKFA
HRMVGYLEEEAIHSYTEFLKELDNGNIENVPAPAIAVDYWRLEADATLRDVVMVVRADEA
HHRDVNHYASDIHYQGHELKEAPAPIGYH
>AtAOX4
MSYRSIYRTLRPVLSSSVQSSGLGIGGFRGHLISHLPNVRLLSSDTSSPVSGNNQPENPI
RTADGKVISTYWGIPPTKITKPDGSAWKWNCFQPWDSYKPDVSIDVTKHHKPSNFTDKFA

YWTVQTLKIPVQLFFQRKHMCHAMLLETVAAVPGMVGGMLLHLKSLRRFEHSGGWIKALL
EEAENERMHLMTFIELSQPKWYERAIVFTVQGVFFNAYFLAYVISPKLAHRITGYLEEEA
VNSYTEFLKDIDAGKFENSPAPAIAIDYWRLPKDATLRDVVYVIRADEAHHRDINHYASD
IQFKGHELKEAPAPIGYH
>AtAOX5
MSQLITKAALRVLLVCGRGNCNMFVSSVSSTSVMKSPYEITAPMRIHDWCGGFGDFKIGS
KHVQGNFNLRWMGMSSASAMEKKDENLTVKKGQNGGGSVAVPSYWGIETAKMKITRKDGS
DWPWNCFMPWETYQANLSIDLKKHHVPKNIADKVAYRIVKLLRIPTDIFFQRRYGCRAMM
LETVAAVPGMVGGMLLHLKSIRKFEHSGGWIKALLEEAENERMHLMTMMELVKPKWYERL
LVMLVQGIFFNSFFVCYVISPRLAHRVVGYLEEEAIHSYTEFLKDIDNGKIENVAAPAIA
IDYWRLPKDATLKDVVTVIRADEAHHRDVNHFASDIRNQGKELREAAAPIGYH
>ZmAOX1
MMSSRAGSILLRHAGSRLFTAAAISPAAASRPLLAGGNGVPAVMLRLMSTSSPAAPTEAK
DEAAKASKVGGDKKAVVINSYWGIEQNNKLARDDGTEWKWTCFRPWETYTADTSIDLTRH
HEPKTLMDKVAYWTVKSLRFPTDIFFQRRYGCRAMMLETVAAVPGMVGGMLLHLRSLRRF
EQSGGWIRALLEEAENERMHLMTFMEVAKPRWYERALVITVQGVFFNAYFLGYLLSPKFA
HRVVGYLEEEAIHSYTEYLKDLEAGKIENVPAPAIAIDYWRLPANATLKDVVTVVRADEA
HHRDVNHFASDIHCQGMQLKQSPAPIGYH
>ZmAOX2
MSTRAAGSALLRHLGPRVFGPVFSPAVAPPRPLLALAGGGERGGALVWVRVRLLSTSAAE
AKEEVAASKGNSGSTAAAKAEAVEAAKEGDGKRDKVVSSYWGVAPSKLMNKDGAEWRWSC
FRPWEAYKPDTTIDLNRHHEPKVLLDKIAYWTVKLLRVPTDIFFQRRYGCRAMMLETVAA
VPGMVGGMLLHLRSLRRFEHSGGWIRALLEEAENERMHLMTFMEVAKPKWYERALVLAVQ
GVFFNAYFLGYLISPKFAHRVVGYLEEEAIHSYTEYLKDLEAGKIENVPAPAIAIDYWQL
PADATLKDVVVVVRSDEAHHRDVNHFASDIHFQGMQLKETPAPIEYH
>ZmAOX3
MSSRMAGAALLRHLGPRLFAAGPAVSGLTARGGMPAAARLLPARMASTAAEAAREDAGAN
KRHGGTEKHEEEAAGGQSKKAVVSYWGIDTPKLVKEDGTEWKWTSFRPWDAYTSDTSIDI
GKHHAPTTLPDKAAYLIVKSLRVPMDLFFQRRHASHALLLETVAAVPGMVGGMLLHLRSL
RRFEHSGGWIRALLEEAENERMHLMTFLEVTQPRWWERALVLTAQGVFFNAYFVGYLLSP
KFAHRVVGYLEEEAVHSYTEYLKDLEAGIIDNTPAPAIAIDYWRLPADAKLKDVVTVVRA
DEAHHRDVNHFASDIHYQGMKLKDTPAPLSYH
>ZmAOX4
MSSRMAGAALLRHLGPRLFAAEPVTGLAARGVMPAAARILPARMSSTAAEAAKEAAAPQQ
RQKPEAAAAAPEGQDKKAVVSYWGIEPRKLVKEDGTEWRWFCFRPWDTYRADTSIDMKKH
HEPKALPDKLAYWLVKSLVVPKQLFFQRRHASHALLLETVAAVPGMVGGMLLHLRSLRRF
EHSGGWIRALLEEAENERMHLMTFLEVAQPKWWERALVLAAQGVYFNAYFVAYLASPKFA
HRFVGYLEEEAVHSYTEYLKDLEAGIIDNTPAPAIAIDYWRLPADARLKDVVAVVRADEA
HHRDVNHFASDIHYQGMKLRDTPAPLGYH
>ZmAOX5
MSSRMAGAALLRHLGPRLFAAEPVTGLAARGVMPAAARILPARMSSTAAEAAKEAAAPQQ
RQKPEAAAAAPEGQDKKAVVSYWGIEPRKLVKEDGTEWRWFCFRPWDTYRADTSIDMKKH
HEPKALPDKLAYWLVKSLVVPKQLFFQRRHASHALLLETVAAVPGMVGGMLLHLRSLRRF
EHSGGWIRALLEEAENERMHLMTFLEVAQPKWWERALVLAAQGVYFNAYFVAYLASPKFA
HRFVGYLEEEAVHSYTEYLKDLEAGIIDNTPAPAIAIDYWRLPADARLKDVVAVVRADEA
HHRDVNHFASVRRGLSGLQESAYFLFGQSELIDRFLWIAGHPLPGHEAEGHACASRLPLI
SQRGLLV
>ZmAOX6
MAVASTSPLSAKPATAPSPPAPGSGLLALGVRRAPATAAWRRLRVEAIRTQRTEVPVEES
APARDAAAAAPLDGNGAGADGSVVPSSDDSWVVKLEQSFNIFATDSVIMVLKGVYGDRYY
ARFFALETIARVPYFAFISVLHLYATFGWWRRADYIKVHFAQSWNEFHHLLIMEELGGDS
LWFDCFLARFMAFFYYFMTVAMYMLSPRMAYHFSECVERHAYSTYDEFLKLHEEELKRLP
APEAALNYYMNEDLYLFDEFQASRTPGSRRPKIDNLYDVFVNIREDEAEHCKTMKTCQTH
GNLRSPHSTPNCLEDDTECVIPENDCEGVVVYKV
>BdAOX1
MSSRMAGSVLLRHAGASRLFSATATSPAAAAARPFLAGGESVPGAWVRLMSTSAGSQAKQ
EAAKAAAPPKDKEGGEKKEVVVNSYWGIEQAKKLVREDGTEWKWSCFRPWETYTADTSID
LTKHHVPKTMLDKIAYWTVKSLRFPTDIFFQRRYGCRAMMLETVAAVPGMVGGMLLHLRS
LRRFEQSGGWIRALLEEAENERMHLMTFMEVAQPRWYERALVIAVQGVFFNAYFFGYLIS
PKFAHRVVGYLEEEAVHSYTEFLKDLEAGKIDDVPAPSIAIDYWRLPANATLKDVVTVVR
ADEAHHRDVNHFASDVYYQGMELKATPAPIGYH
>BdAOX2
MPSWQWWHVVARRHVVPLAPEKLARLQGRERAATSSPSRATHRREGSSSSAMSSRVAGPA
VLLRHLGRRIFSSPVSPASPVAAQRPLLSGGEGAVWARLRLLSTSAAEEAAKEEAAASKE
NSASTAAAKAEAAQAAKDGDKTVVSSYWGIVPAKLVNKDGAEWKWSCFRPWEAYTSDTTI
DLKKHHEPKVLLDKIAYWTVKSLRVPTDIFFQRRYGCRAMMLETVAAVPGMVGGMLLHLR
SLRRFEHSGGWIRALLEEAENERMHLMTFMEVAGPKWYERALVLAVQGVFFNAYFLGYLL
SPKFAHRVVGYLEEEAVHSYTEFLRDIEAGKIDNVPAPRIAIDYWRLPPDATLRDVVVVV
RADEAHHRDVNHFASDIHFQGLELNKTPAPLGYH
>BdAOX3
MPSWQWWHVVARRHVVPLAPEKLARLQGRERAATSSPSRATHRREGSSSSAMSSRVAGPA
VLLRHLGRRIFSSPVSPASPVAAQRPLLSGGEGAVWARLRLLSTSAAEEAAKEEAAASKE
NSASTAAAKAEAAQAAKDGDKTVVSSYWGIVPAKLVNKDGAEWKWSCFRPWEAYTSDTTI

DLKKHHEPKVLLDKIAYWTVKSLRVPTDIFFQRRYGCRAMMLETVAAVPGMVGGMLLHLR
SLRRFEHSGGWIRALLEEAENERMHLMTFMEVAGPKWYERALVLAVQGVFFNAYFLGYLL
SPKFAHRVVGYLEEEAVHSYTEFLRDIEAGKIDNVPAPRIAIDYWRLPPDATLRDVVVVV
RADEAHHRDVNHFASGWSSTRRLPRSDTTDD
>BdAOX4
MSSRMAGATLLRHLGPRLFAAAEPASGLAARSIMAPAAARILPARMASTASAAPDAKEGA
SAAAKTDSAATPEQSKTKSVVSYWGIESRKLVKPDGTEWPWFCFTPWDTYRADTSIDMEK
HHKPKSVPDKVAYYAVRSLRVPMDLFFQRRHASHALLLETVAAVPPMVGGMLLHLRSLRR
FEHSGGWIRALMEEAENERMHLMTFLEVTQPNWWERALVMAAQGVFVNAYFVGYLVSPKF
AHRFVGYLEEEAVHSYTEYLKDLEAGKIENTPAPAIAIDYWRLPADATLKDVVTVIRADE
AHHRDANHYASDIHYQGLTLKETPAPIGYH
>BdAOX5
MSSRMAGATLLRHLAPRLVAAAEPASGLAARSIMPAAARIFPARMASTAAAPDVQEGAAG
ATGKTEGQSKTKAVVSYWGIEPRKLVKADGTEWPWFCFRPWDTYTADTAIDMQKHHEPKS
LPDKIAYYTVKTLGVPKDLFFQRRHASHALLLETVAAVPPMVGGMLLHLRSLRRFEHSGG
WIRALMEEAENERMHLMTFLEVTQPKWWERALVMAVQGVFFNAYFVGYLVSPKFAHRFVG
YLEEEAVKSYTEYLKDLEAGKIENTPAPAIAIDYWRLPADATLKDVVAVVRADEAHHRDA
NHYASDIHYQGLTLKETPAPIGYH
>BdAOX6
MAVASVSASASHLPAALSSNPSPRSPPSFLPTPLRAGRLSAATGGLGLSPAVLLLTRKNG
RRFPLGATARTQREKAELETDVAAVEESFPVRETGAPEGAADNDDDGALPPAEDVGWVVR
LEQSFNIFLTDSIITILDGLYGDRNYARFFVLETIARVPYFAFISVLHMYETFGWWRRAD
YIKVHFAESMNEFHHLLIMEELGGNSELVDRFLARFSAFFYYFMTVAMYMLSPRMAYHFS
ECVERHAYSTYDKFLKLNGEELKKLPAPEAAINYYMNEDLYLFDEFQTSRVPCSRRPKVD
NLYDVFVNIRDDEAEHCKTMKACQTHGNLRSPHSMERRVDETDAECVVPENDCEGIMDCV
KKSLTSED
>HvAOX1
MSSRMAGSVLLRHAGAGAGAGRLFATTASPAARTALAGGEGAWARMMSTSAASHAKDEAAKA
AATGDGEKKEVAVNSYWGIEQSKKLVREDGTEWKWSCFRPWETYTADTSIDLTKHHVPNT
MLDKIAYYTVKSLRFPTDIFFQRRYGCRAMMLETVAAVPGMVGGMLLHLRSLRRFEQSGG
WIRALLEEAENERMHLMTFMEVAQPRWYERALVITVQGVFFNAYFFGYLISPKFAHRVVG
YLEEEAVHSYTEFLKDLDDGKIDNVPAPAIAIDYWRLPANATLKDVVTVVRADEAHHRDV
NHFASDVYYQGMELKATPAPIGYH
>HvAOX2
MPSWRALARRHRHVVPSPSQSSARLQLREAATTSFASRAAAHEAGSSSSAMSSRVAGSVL
LRHLGPRVFGPAAAAAQRPLLAGGEGGAVAVRARPLSTSAAEAAREEAAASKDNVASTAA
ATAEAMQAAKAEAVQAAKGGKSPAVSSYWGIVPAKLVNKDGAEWKWSCFRPWEAYTSDTT
IDLTKHHKPKVLLDKIAYWTVKSLRVPTDIFFQRRYGCRAMMLETVAAVPGMVGGMLLHL
RSLRRFEQSGGWIRALLEEAENERMHLMTFMEVANPKWYERALVLAVQGVFFNAYFVGYI
LSPKFAHRVVGYLEEEAIHSYTEFLRDLEAGRIDNVPAPRIAIDYWRLPADARLKDVVTV
VRADEAHHRDVNHFAADIHFQGLELNKTPAPLGYH
>HvAOX3
MSSRMAGATLLRHLGPRLFAAAEPASGLAASARGVMPAATRIFPARMASTAAAPHAKQEE
ATEKPQGATTPEQAVSYWGIEPRKLVKDDGTEWPWFCFRPWDTYRPDTSIDVAKHHEPRA
LPDKVAYLIVRTLRAGSDLFFQRRHASHALLLETVAAVPPMVGGVLLHLRSLRRFEHSGG
WIRALMEEAENERMHLMTFMEVTQPLWWERALVLATQGVFFNAYFVGYLVSPKFAHRFVG
YLEEEAVHSYTEYLKDLEAGLIENTPAPAIAIDYWRLPADARLKDVVTAVRADEAHHRDA
NHYASDIHYQGMTLNQTPAPLGYH
>HvAOX4
MSSRMAGATLLRHLGPRLFVAAEPASGLAAGARGIMPAAARIFPARMASTEAAAPHAKQE
DDAKSPQAAATPAQQNKKAVVSYWGIEPRKLVKDDGTEWPWFCFRPWDTYRPDTSIDVAK
HHEPRALPDKVAYFVVRSLRVPRDLFFQRRHASHALLLETVAAVPPMVGGVLLHLRSLRR
FEHSGGWIRALMEEAENERMHLMTFMEVTQPRWWERALVLAAQGVFFNAYFVGYLISPKF
AHRFVGYLEEEAVESYTEYLKDLEAGLIENTPAPAIAIDYWRLPADARLKDVVTAVRADE
AHHRDANHYASDIHYQGMTLNQTPAPLGYH
>GmAOX1
MKNVLVRSAARALLGGGGRSYYRQLSTAAIVEQRHQHGGGAFGSFHLRRMSTLPEVKDQH
SEEKKNEVNDTSNAVVTSYWGITRPKVRREDGTEWPWNCFMPWDSYHSDVSIDVTKHHTP
KSLTDKVAFRAVKFLRVLSDIYFKERYGCHAMMLETIAAVPGMVGGMLLHLKSLRKFQHS
GGWIKALLEEAENERMHLMTMVELVKPSWHERLLIFTAQGVFFNAFFVFYLLSPKAAHRF
VGYLEEEAVISYTQHLNAIESGKVENVPAPAIAIDYWRLPKDATLKDVVTVIRADEAHHR
DVNHFASDIHHQGKELKEAPAPIGYH
>GmAOX2
MKLTALNSTVRRALLNGRNQNGNRLGSAALMPYAAAETRLLCAGGANGWFFYWKRTMVSP
AEAKLPEKEKEKEKAKAEKSVVESSYWGISRPKVVREDGTEWPWNCFMPWESYRSNVSID
LTKHHVPKNVLDKVAYRTVKLLRIPTDLFFKRRYGCRAMMLETVAAVPGMVGGMLLHLRS
LRKFQQSGGWIKALLEEAENERMHLMTMVELVKPKWYERLLVLAVQGVFFNAFFVLYILS
PKVAHRIVGYLEEEAIHSYTEYLKDLESGAIENVPAPAIAIDYWRLPKDARLKDVITVIR
ADEAHHRDVNHFASDIHFQGKELREAPAPIGYH
>GmAOX3
MMMMMSRSGANRVANTAMFVAKGLSGEVGGLRALYGGGVRSESTLALSEKEKIEKKVGLS
SAGGNKEEKVIVSYWGIQPSKITKKDGTEWKWNCFRPWETYKADLSIDLEKHHAPTTFLD
KMAFWTVKVLRYPTDVFFQRRYGCRAMMLETVAAVPGMVAGMLLHCKSLRRFEHSGGWIK

ALLEEAENERMHLMTFMEVAKPKWYERALVITVQGVFFNAYFLGYLLSPKFAHRMVGYLE
EEAIHSYTEFLKELDKGNIENVPAPAIAIDYWQLPPGSTLRDVVMVVRADEAHHRDVNHF
ASDIHYQGRELREAAAPIGYH
>GmAOX4
MKLIALSSTVRRALLNGRNQNCNRLGSAAETRLLCAGGANGGFFYWKRTMASPAEAKFPE
KEKAEAEKSVVESSYWGISRPKVSREDGTEWPWNCFMVRILFLIRNQALGDLSSNVSIDL
TKHHVPKNFLDKVAYRTVKLLRIPTDLFFKRRYGCRAMMLETVAAVPGMPSGGWIKALLE
EAENERMHLMTMVELVKPKWYERLLVLAVQGVFFNAFFVLYILSPKVAHRIVGYLEEEAI
HSYTEYLKDLESGAIENVPAPAIAIDYWRLPKDAKLKDVVTVIRADEAHHRDVNHFASDI
HFQGKELRDAPAPVGYH
>GmAOX5
MAAIFSSSLFASSPLPKPLSSSYSRIHTAPQLFRARSSLLQDNEKKVIVHDSFPSKTSPL
HTADKSTGGNSINTSAFEKRIIKDSVIKILDTLYHDRHYARFFVLETIARVPYFAFMSVL
HMYESFGWWRRADYLKVHFAESWNEMHHLLIMEELGGNAWWFDRFLAQHIAIFYYIMTVL
MYAVSPRMAYHFSECVESHAFETYDKFIKVQGDELKKMPAPEVAVNYYTGDDLYLFDEFQ
TSRVPNSRRPKIENLYDVFVNIRDDEAEHCKTMKACQTHGNLRSPHSYAEDDDSSVCALE
ADCEGIVDCIKKSVASNPAKVK
>GmAOX6
MAAIFSSSLFVSSPLPKPLSSSYSRIHKAPQLFRARSSLLQDKEEKVIVQDSFPSKTSPL
HSADKSTSGNSINTSAFEKRIIKVEQSVNIFLTDSVIKILDTLYHDRHYARFFVLETIAR
VPYFAFMSVLHMYESFGWWRRADYLKVHFAESWNEMHHLLIMEELGGNAWWFDRFLAQHI
AIFYYIMTVLMYAVSPRMAYHFSECVESHAFETYDKFIKVQGDELKKMPAPEVAVNYYTG
DDLYLFDEFQTSRVPNSRRPKIENLYDVFVNIRDDEAEHCKTMKACQTHGNLRSPHSYAE
DDDSSVCALEADCEGIVDCIKKSVTSNAAKVK
>GmAOX7
MAAIFSSSLFASSPLPKPLSSSYSRIHTAPQLFRARSSLLQDNEKKVIVHDSFPSKTSPL
HTADKSTGGNSINTSAFEKRIIKVEQSVNIFLTDSVIKILDTLYHDRHYARFFVLETIAR
VPYFAFMSVLHMYESFGWWRRADYLKVHFAESWNEMHHLLIMEELGGNAWWFDRFLAQHI
AIFYYIMTVLMYAVSPRMAYHFSECVESHAFETYDKFIKVQGDELKKMPAPEVAVNYYTG
DDLYLFDEFQTSRVPNSRRPKIENLYDVFVNIRDDEAEHCKTMKACQTHGNLRSPHSYAE
DDDSSVCALEADCEGIVDCIKKSVASNPAKVK
>GmAOX8
MAAIFSSSLFASSPLPKPLSSSYSRIHTAPQLFRARSSLLQDNEKKVIVHDSFPSKTSPL
HTADKSTGGNSINTSAFEKRIIKVEQSVNIFLTDSVIKILDTLYHDRHYARFFVLETIAR
VPYFAFMSVLHMYESFGWWRRADYLKVHFAESWNEMHHLLIMEELGGNAWWFDRFLAQHI
AIFYYIMTVLMYAVSPRMAYHFSECVESHAFETYDKFIKVQGDELKKMPAPEVAVNYYTG
DDLYLFDEFQTSRVPNSRRPKIENLYDVFVNIRDDEAEHCKTMKACQTHGNLRSPHSYAE
DDDSSVCALEADCEGIVDCIKKSVASNPAKMLAKNFLAGNIIVTFC
>GmAOX9
MRRKCRLLTKSCESTWTTHLLVFPLSSLQFNTHTQSSLLSKHGGNFLFFTLRFLASSQTT
FFLLFSNPYRSTTFQDNEKKVIVHDSFPSKTSPLHTADKSTGGNSINTSAFEKRIIKVEQ
SVNIFLTDSVIKILDTLYHDRHYARFFVLETIARVPYFAFMSVLHMYESFGWWRRADYLK
VHFAESWNEMHHLLIMEELGGNAWWFDRFLAQHIAIFYYIMTVLMYAVSPRMAYHFSECV
ESHAFETYDKFIKVQGDELKKMPAPEVAVNYYTGDDLYLFDEFQTSRVPNSRRPKIENLY
DVFVNIRDDEAEHCKTMKACQTHGNLRSPHSYAEDDDSSVCALEADCEGIVDCIKKSVAS
NPAKVK
>MtAOX1
MMMRHGGAMNTAMMFAKKGLLGGEVGVPNKWGYLVRSTPLVRKTSTFTANLSDQKDNKNV
DKTPPSSQGGAGDNKDEKGITSYWGVQPSKITKPDGTEWKWNCFRPWETYKADVTIDLTK
HHKPTTFLDKMAYWTVKSLRWPTDIFFQRRYGCRAMMLETVAAVPGMVGGMLLHCKSLRR
FEQSGGWIKALLEEAENERMHLMTFMEVAKPKWYERALVITVQGVFFNAYFLGYLLSPKF
AHRMVGYLEEEAIHSYTEFLKELDKGNIENVPAPAIAIDYWQLPQNSTLRDVVEVVRADE
AHHRDVNHFASDIHYQGRELREAAAPIGYH
>MtAOX2
MRNILLRSTARALFRNGGNYHRSFSTAVIVQPRHHQHGGGACGNLYWQRMSTLPEKKDQQ
TEESKKDANHNAVVSSYWGISRPKVLKEDGTEWPWNCFMPWESYSSDVSIDVTKHHVPKT
FGDKFAFRSVKFLRVLSDLYFKERYGCHAMMLETIAAVPGMVGGMLLHLKSLRKFQHAGG
WIKALLEEAENERMHLMTMVELVKPSWHERLLVITAQGVFFNGFFVFYILSPKIAHRFVG
YLEEEAVISYTQYLNAIESGKVENVPAPAIAIDYWRLPNDATLKDVVTVIRADEAHHRDV
NHFASDIHHQGKELKEAPAPVGYH
>MtAOX3
MKNSLLRSTARALFHSSRNYHCSFSTAVIVQPRHQNGGGTRGSFYWQKMSTLPEKKDQHS
EENKNSNDSNTVVSSYWGITRPKVKREDGTEWPWNCFMPWESYSSDVSIDVTKHHVPKTF
GDKFAFRSVKFLRVLSDLYFKERYGCHAMMLETIAAVPPMVGGMLLHLKSLRKFQHTGGW
IKALLEEAENERMHLMTMVELVKPSWHERLLVITAQGVFFNAFFVFYILSPKTAHRFVGY
LEEEAVISYTQHLNAIESGKVENVPAPAIAIDYWRLPKDATLKDVITVIRADEAHHRDVN
HFASDIHHQGKELKEAPAPIGYH
>MtAOX4
MKHSALCYVARRALIGGRNSNRQSSAVVRSFAAAEIGQKHLYADGGNGGLFYWKRMMSSQ
AAPSKPSAEETEAKSTEKNEKKKEESSGTKNNVVASSYWGISRPKIMREDGTEWPWNCFM
PWETYQSNVSIDLNKHHVPKNFLDKVAYRTVKLLRIPTDVFFKRRYGCRAMMLETVAAVP
GMVGGMLLHLKSLRKFQHSGGWVKALLEEAENERMHLMTMVELVKPKWYERFLVLAVQGV
FFNAFFVLYILSPKVAHRVVGYLEEEAIHSYTEYLKDIDSGAIENVPAPAIAIDYWRLPK

DAKLKDVITVIRADEAHHRDVNHFASDIHFHGKELRDAPAPLGYH
>MtAOX5
MASTAMFSSSLFPITPLNKLSSSRNSLIFRPLSFRPPLFRIRSSLLQDKEDKVITQNTFP
SKTSPLDSVTENSTNDDDTSSTSAWEKGVIKVEQSVNIFLTDSVIKILDALYRDRNYARF
FVLETIARVPYFAFMSILHMYESFGWWRRADYLKVHFAESWNEMHHLLIMEELGGNAWWF
DRFLAQHIAIFYYFMTALMYLISPRMAYHFSECVESHAFETYDKFIKEQGEELKKMPAPE
VAVNYYTGGDLYLFDEFQTSRVPNTRRPTIDNLYDVFLNIRDDEAEHCKTMRACQTYGNL
RSPHSYADAEDDDESVCTIEAGCEGIVDCIKKSVTSNPAKVK
>SbAOX1
MSTRAAGSVLLRHLGPRVFGSVSSSPAAAPPRPLLALAGGGEWRGAAVWVRLLSTSAAEA
KEEAAASKGNAGSTAAAKAEAVEAAKEGDGKKDKVVSSYWGVAPSKLMSKDGVEWRWSCF
RPWEAYKPDTSIDLTRHHEPKVLLDKIAYWTVKLLRVPTDIFFQRRYGCRAMMLETVAAV
PGMVGGMLLHLRSLRRFEHSGGWIRALLEEAENERMHLMTFMEVAKPKWYERALVLAVQG
VFFNAYFLGYLISPKFAHRVVGYLEEEAIHSYTEYLKDLEAGKIENVPAPAIAIDYWQLP
ADATLKDVVVVVRSDEAHHRDVNHFASDIHFQGMQLKETPAPIEYH
>SbAOX2
MSSRMAGAAVLRHLGPRLFAAEQAAVSGLTARGVMPAAARLLPARMASTAAEAAREDAGA
KQHGGTGKQEEAAGGGQSKKAIVSYWGIEAPKLVKEDGTEWKWTSFRPWDAYTSDTSIDV
KKHHAPTTLPDKAAYLIVKSLRVPMDLFFQRRHASHALLLETVAAVPGMVGGMLLHLRSL
RRFEHSGGWIRALLEEAENERMHLMTFLEVTQPKWWERALVLATQGVFFNAYFVGYLLSP
KFAHRVVGYLEEEAVYSYTEYLKDLEAGIIENTPAPAIAIDYWRLPADAKLKDVVTVVRA
DEAHHRDVNHFASDIHYQGMKLRDTPAPLGYH
>SbAOX3
MSSRMAGAAVLRHLGPRLFAAEPLTVPAAARILPARMSSTAAAPEQRQKPEAPAPEGQDK
IKAVVSYWGIQPRKLVKEDGTEWRWFCFRPWDTYRADTSIDMKKHHEPKALPDKLAYWLV
KSLIVPKQLFFQRRHASHALLLETVAAVPGMVGGMLLHLGSLRRFEHSGGWIRALLEEAE
NERMHLMTFLEVAQPKWWERALVLAAQGVFFNAYFVAYLASPKFAHRFVGYLEEEAVHSY
TEYLKDLEAGVIENTPAPAIAIDYWRLPADAKLKDVVTVVRADEAHHRDVNHFASDIHYQ
GMKLKDTPAPLGYH
>SbAOX4
MAVASTSPLSAKPTTATSPPTPAFGFLAPRSRRGRATAWRSLLVEAIRTQREKQGTEGHF
EESAAAPLDGVGVGADDPVVPSSDASDWVVKLEQSFNIFATDSVIMVLKGVYRDRYYARF
FALETIARVPYFAFISVLHMYSTFGWWRRADYIKVHFAQSWNEFHHLLIMEELGGNSLWI
DCFLARFMAFFYYFVTVAMYMLSPRMAYHFSECVERHAYSTYDEFLKLHEEELKRLPAPE
AALEYYLNEDLYLFDEFQASRSPGSRRPKIDNLYDVFVNIRDDEAEHCKTMKTCQTHGNL
RSPHSTQNCLEADTECVIPENDCEGARVQKAL
>SbAOX5
MAVASTSPLSAKPTTATSPPTPAFGFLAPRSRRGRATAWRSLLVEAIRTQREKQGTEGHF
EESAAAPLDGVGVGADDPVVPSSDASDWVVKLEQSFNIFATDSVIMVLKGVYRDRYYARF
FALETIARVPYFAFISVLHMYSTFGWWRRADYIKVHFAQSWNEFHHLLIMEELGGNSLWI
DCFLARFMAFFYYFVTVAMYMLSPRMAYHFSECVERHAYSTYDEFLKLHEEELKRLPAPE
AALEYYLNEDLYLFDEFQASRSPGSRRPKIDNLYDVFVNIRDDEAEHCKTMKTCQTHGNL
RSPHSTQNCLEADTECVIPENDCEGIVDCVKKSLTK
>SbAOX6
MCCSFPQDSVIMVLKGVYRDRYYARFFALETIARVPYFAFISVLHMYSTFGWWRRADYIK
VHFAQSWNEFHHLLIMEELGGNSLWIDCFLARFMAFFYYFVTVAMYMLSPRMAYHFSECV
ERHAYSTYDEFLKLHEEELKRLPAPEAALEYYLNEDLYLFDEFQASRSPGSRRPKIDNLY
DVFVNIRDDEAEHCKTMKTCQTHGNLRSPHSTQNCLEADTECVIPENDCEGIVDCVKKSL
TK
>SbAOX7
MCYYSFPQESMIMILDGVYRDRNYARFFVLETIARVPYFGFISVLHLYETFGWWRRADYI
KVHFAQSWNEFHHLLIMEELGGNALWIDRFLARFMAFFYYFMTVAMYMLSPRMAYHFSEC
VERHAYSTYDKFLKLHEEELKRLPAPEAALNYYLNEDLYLFDEFQTAGVPCSRRPKIDNL
YDVFVNVRDDEAEHCKTMKACQTHGNLRSPHSMLDCLEVDSECVIPEPEDDCEGFVDSVK
KSLTK
>SbAOX8
MAVASASPLPSAPSAKPATAPSPLAPSSGFLALRARRCGAPRLRPAAAAWRRLRVEAIRT
QQEKQRTEVPVEESAPVRGAAAPLDGVGADDPMVASSEESWVVRLEQSFNIFATESMIMI
LDGVYRDRNYARFFVLETIARVPYFGFISVLHLYETFGWWRRADYIKVHFAQSWNEFHHL
LIMEELGGNALWIDRFLARFMAFFYYFMTVAMYMLSPRMAYHFSECVERHAYSTYDKFLK
LHEEELKRLPAPEAALNYYLNEDLYLFDEFQTAGVPCSRRPKIDNLYDVFVNVRDDEAEH
CKTMKACQTHGNLRSPHSMLDCLEVDSECVIPEPEDDCEGFVDSVKKSLTK
>ScAOX1
MSSRMAGSVLLRHAGAGASRLFATTATASPAARTALAGGDGAWVRLMSTSAASQAKDQAA
KVAAAAEAAKGDGEKKEVAINSYWGIEQSKKLVREDGTEWKWSCFRPWETYTADTSIDLT
KHHVPNTMLDKIAYYTVKSLRFPTDIFFQRRYGCRAMMLETVAAVPGMVGGMLLHLRSLR
RFEQSGGWIRALLEEAENERMHLMTFMEVAQPRWYERALVIAVQGVFFNAYFFGYLISPK
FAHRVVGYLEEEAVHSYTEFLKDLDDGKIDNVPAPAIAIDYWRLPANATLKDVVTVVRAD
EAHHRDVNHFASDVYYQGMQLKATPAPIGYH
>ScAOX2
MSSRMAGATLLRHLGPRLFAAAEPAYGLAASARGIMPAAARIFPARMASTVAGPHAKQEG
DAEKPQGATTPEQNKKAVASYWGIEPRKLVKDDGTEWPWFCFRPWDTYRPDTSIDVAKHH
EPRAVADKVAYFIVRTLRAGSDLFFQRRHASHALLLETVAAVPPMVGGVLLHLRSLRRFE

HSGGWIRALMEEAENERMHLMTFMEVTQPLWWERALVLATQGVFFNAYFVGYLVSPKFAH
RFVGYLEEEAVHSYTEYLKDLEAGLIENTPAPAIAIDYWRLPADARLKDVVIAVRADEAH
HRDANHYASDIHYQGMTLNQTPAPLGYH
>ScAOX3
MSSRMAGATLLRHLGPRLFAAAEPASGLAASARGIMPAAARIFPARMASTEAAAPRAKQE
EDAEKPQEAATPEQQSKKPVVSYWGIEPRKLVKDDGTEWPWFCFRPWDTYRPDTSIDVTK
HHEPKALADKVAYFVVRSLRVPRDLFFQRRHASHALLLETVAAVPPMVGGVLLHLRSLRR
FEHSGGWIRALMEEAENERMHLMTFMEVTQPRWWERALVLAAQGVFFNAYFVGYLISPKF
AHRFVGYLEEEAVESYTEYLKDLEAGLIENTPAPAIAIDYWRLPADARLKDVVTAVRADE
AHHRDANHYASDIHYQGMTLNQTPAPLGYH
>TaAOX1
MSSRMAGSVLLRRAGAGASRLFATTPTSPAARTALAGGDGAWVRMMSTSAASQVKDEAAK
AVKAEAAKGDGEKKEVAISSYWGIEQSKKLVREDGTEWKWSCFRPWETYTADTSIDLTKH
HVPNTMLDKIAYYTVKSLRFPTDIFFQRRYGCRAMMLETVAAVPGMVGGMLLHLRSLRRF
EQSGGWIRALLEEAENERMHLMTFMEVAQPRWYERALVIAVQGVFFNAYFFGYLISPKFA
HRVVGYLEEEAVHSYTEFLKDLDDGKIDNVPAPAIAIDYWRLPANATLKDVVTVVRADEA
HHRDVNHFASDVYYQGMQLKATPAPIGYH
>TaAOX2
MSSRMAGSVLLRRAGAGAGRLFATTASPAARTALGGGEGAWVRMMSTSAASQVKDEAAKG
VKAEAAKGDGEKKEVAISSYWGIEQSKKLVREDGTEWKWSCFRPWETYTADTSIDLTKHH
VPNTMLDKIAYYTVKSLRFPTDIFFQRRYGCRAMMLETVAAVPGMVGGMLLHLRSLRRFE
QSGGWIRALLEEAENERMHLMTFMEVAQPRWYERALVIAVQGVFFNAYFFGYLISPKFAH
RVVGYLEEEAVHSYTEFLKDLDDGKIDNVPAPAIAIDYWRLPANATLKDVVTVVRADEAH
HRDVNHFASDVYYQGMQLKATPAPIGYH
>TaAOX3
MSSRMAGSVLLRRAGAGASRLFATTPSPAARAVLGGGEGAWVRLMSTSAASQVKDEAAKA
VKAEAAKAVKAEAAKGDGEKKEVAISSYWGIEQSKKLVREDGTEWKWSCFRPWETYTADT
SIDLTKHHVPNTMLDKIAYYTVKSLRFPTDIFFQRRYGCRAMMLETVAAVPGMVGGMLLH
LRSLRRFEQSGGWIRALLEEAENERMHLMTFMEVAQPRWYERALVIAVQGVFFNAYFFGY
LISPKFAHRVVGYLEEEAVHSYTEFLKDLDDGKIDNVPAPAIAIDYWRLPANATLKDVVT
VVRADEAHHRDVNHFASDVYYQGMQLKATPAPIGYH
>TaAOX4
MSSRVAGSVLLRHLGPRVFGPTTPAAQRPLLAGGEGGAVVVWARPLSTSAAEAAREEAAA
SKDNVASTAAATAEAMQAAKAAKEGGKSPVSSYWGIVPAKLVNKDGAEWKWSCFRPWEAY
TSDTTIDLTKHHKPKVLLDKIAYWTVKSLRVPTDIFFQRRYGCRAMMLETVAAVPGMVGG
MLLHLRSLRRFEQSGGWIRALLEEAENERMHLMTFMEVAKPKWYERALVLAVQGVFFNAY
FLGYIVSPKFAHRVVGYLEEEAIHSYTEFLRDLEAGRIENVPAPRIAIDYWRLPADARLK
DVVTVVRADEAHHRDVNHFAADIHFQGLELNKTPAPLGYH
>TaAOX5
MPSWRALARRHRHVIPSPSRSLARPQVLDPATTSFASRAAAHQAGSPSSAMSSRVAGSVL
LRHLGPRVFGPTTQAAQRTLLAGGEGGAVAMWAWPLSTSAAEAAREEAAASKDNVASTAA
ATAEAMQAAKAEAVQAAKEGGKSPASSYWGIVPAKLVNKDGAEWKWSCFRPWEAYTSDTT
IDLTKHHKPKVLLDKIAYWTVKSLRVPTDIFFQRRYGCRAMMLETVAAVPGMVGGMLLHL
RSLRRFEQSGGWIRALLEEAENERMHLMTFMEVANPKWYERALVLAVQGVFFNAYFLGYI
VSPKFAHRVVGYLEEEAIHSYTEFLRDLEDGRIENVPAPRIAIDYWRLPPDARLKDVVTV
VRADEAHHRDVNHFAADIHFQGLELNKTPAPLGYH
>TaAOX6
MPSWRALARRQRHVIPSPSQSLARPQVLEPATTSFASRAAAHQAGSSSSAMSSRVAGSVL
LRHLGPRVFGPTTPAAQRPLLAGGEGGAVAVAMWARPLSTSAAEAAREEATASKDNVAST
AAATAEAMQAAKADAVQAAKEGKSPAASSYWGIVPAKLVNKDGAEWKWSCFRPWEAYTSD
TTIDLSKHHKPKVLLDKIAYWTVKSLRVPTDIFFQRRYGCRAMMLETVAAVPGMVGGMLL
HLRSLRRFEQSGGWIRALLEEAENERMHLMTFMEVANPKWYERALVLAVQGVFFNAYFLG
YIVSPKFAHRVVGYLEEEAIHSYTEFLRDLEAGRIENVPAPRIAIDYWRLPADARLKDVV
TVVRADEAHHRDVNHFAADIHFQGLELNKTPAPLGYH
>TaAOX7
MSSRMAGATLLRHLGPRLFAAAEPASGLAASARGIMPAAARIFPARMASTEAAGPRAKQE
EATEKPQGATTPEQNKKAVVSYWGIEPRKLVKDDGTEWPWFSFRPWDTYRPDTSIDVAKH
HEPRAVADKVAYLIVRTLRKGSDLFFQRRHASHALLLETVAAVPPMVGGVLLHLRSLRRF
EHSGGWIRALMEEAENERMHLMTFMEVTQPLWWERALVLATQGVFFNAYFVGYLISPKFA
HRFVGYLEEEAVHSYTEYLKDLEAGLIENTPAPAIAIDYWRLPADARLKDVVIAVRADEA
HHRDANHYASDIHYQGMTLNQTPAPLGYH
>TaAOX8
MSSRMAGATLLRHLGPHLFAAAEPASGLAASARGILPAAARIFPARMASTAAGAHAKQEG
DAEKPESATAPEQNKKPVASYWGIEPRKLVKDDGTEWPWFSFRPWDTYRPDTSIDVAKHH
EPRAVADKVAYLIVRTLRAGSDLFFQRRHASHALLLETVAAVPPMVGGVLLHLRSLRRFE
HSGGWIRALMEEAENERMHLMTFMEVTQPLWWERALVLATQGVFFNAYFVGYLISPKFAH
RFVGYLEEEAVHSYTEYLKDLEAGLIENTPAPAIAIDYWRLPADARLKDVVIAVRADEAH
HRDANHYASDIHYQGMTLNQTPAPLGYH
>TaAOX9
MSSRMAGATLLRHLGPRLFAAAEPASGLAASARGIMPAAARIFPARMASTEAAAPHAKQE
DDAASPQAAATPEQQNKKPVVSYWGIEPRKLVKDDGTEWPWFCFRPWDTYRPDTSIEVAK
HHEPKALADKVAYFVVRSLRVPRDLFFQRRHASHALLLETVAAVPPMVGGVLLHLRSLRR
FEHSGGWIRALMEEAENERMHLMTFMEVTQPRWWERALVLAAQGVFFNAYFVGYLISPKF

AHRFVGYLEEEAVESYTEYLKDLEAGLIENTPAPAIAIDYWRLPADARLKDVVTAVRADE
AHHRDANHYASDVHYQGMTLNQSPAPLGYH
>TaAOX10
MSSRMAGATLLRHLGPRLFAAAEPASGLAASARGIMPAAARIFPARMASTEAAAPHAKQE
DDAGTPQAAATPEQQSKKAVVSYWGIEPRKLVKEDGTEWPWFCFRPWDTYRPDTSIDVTK
HHEPKALADKVAYFVVRSLRVPRDLFFQRRHASHALLLETVAAVPPMVGGVLLHLRSLRR
FEHSGGWIRALMEEAENERMHLMTFMEVTQPRWWERALVLAAQGVFFNAYFVGYLISPKF
AHRFVGYLEEEAVESYTEYLKDLEAGLIENTPAPAIAIDYWRLPADARLKDVVTAVRADE
AHHRDANHYASDIHYQGMTLNQTPAPLGYH
>TaAOX11
MSSRMAGATLLRHLGPRLFAAAEPASRLAASARGIMPAAARVFPARMASTEAAGPRAKHE
EATEKPQGATAPEQNKKAVPSYWGIEPRKLVKDDGTEWPWFSFRPWDTYRPDTSIDVAKH
HEPRAVADKVAYLIVRTLRKGSDLFFQRRHASHALLLETVAAVPPMVGGVLLHLRSLRRF
EHSGGWIRALMEEAENERMHLMTFMEVTQPLWWERALVLATQGVFFNAYFVGYLVSPKFA
HRFVGYLEEEAVHSYTEYLKDLEAGLIENTPAPAIAIDYWRLPADARLKDVVTAVRADEA
HHRDANHYASDIHYQGMTLNQTPAPLGYH
>TaAOX12
MAGATLLRHLGPRLFAAAEPASGLAASARGIMPAAARIFPARMASTEAAAPHAKQEDDAA
SPQAAATPEQQNKKPVVSYWGIEPRKLVKDDGTEWPWFCFRPWDTYRPDTSIDVTKHHLP
KALADKVAYFVVRSLRVPRDLFFQRRHASHALLLETVAAVPPMVGGVLLHLRSLRRFEHS
GGWIRALMEEAENERMHLMTFMEVTQPRWWERALVLAAQGVFFNAYFVGYLISPKFAHRF
VGYLEEEAVESYTEYLKDLEAGLIENTPAPAIAIDYWRLPADARLKDVVTAVRADEAHHR
DANHYASDIHYQGMTLNQTPAPLGYH
>TaAOX13
MSSRMAGATLLRHLGPRLFTAADPASGLAASARGIMPAAARIFPARMASTEAAAPHAKQE
EATEKPQGATTPEHNKKAVVSYWGIEPRKLVKDDGTEWTWFSFRPWDTYRPDTSIDMAKH
HEPRAVADKVAYLIVRTLRAGSDLFFQRRHASHALLLEMVAAVPPMVGGVLLHLRSLRRF
EHSSGWIRALMEEAENERMHLMTFMEVTQPLWWERALVLATQGVFFNAYFVGYLVSPKFA
HRFVGYLEEEAVHSYTKYLKDLEAGLIENTPAPAIAIDYWRLPADARLKDVVTAVRADEA
HHRDANHYASDIHYQGMTLNQTPAPLGYH
>TaAOX14
MAMVQSVARRGARSVATAQLGMHLFSVSCCSPAALGVGTARTAATLKKGEKEAASYWGVA
PARLVKEDGTEWKWPCFRPWDAYEADVSIDLKKQWRTQGVCQKHHRPATLGDKVALWTVK
AMRWPTDLFFQSLRRFEQSGGWIRPLLEEAENERMHLMTFMEVSQPRWYERALVVAVQGV
FFHAYLATYLASPKVAHRMVGYLEEEAVHSYTEFLRDLEAGKIDDVPAPTRRTTGTSNHY
ASDIHCQGHALREVAAPIGYH
>TaAOX15
MAASAAPLRAALARSHAAASARAPTAFLPLPPRARRLNPAVLASGRGRRIRAEATARTRQ
EKEQQEAEVSAVEDSFAVREAAAAPPPEEEGGFDEELTLAGEDGDWVVRFEQSFNVFLTD
TVIFILDILYRDRDYARFFVLETIARVPYFAFISVLHLYETFGWSRRADNIKVHFAESMN
EFHHLLIMEALGGNSVWIDRFLARFSAFFYYFVTVAMYMLSPRMAYHFSECVERHAYSTY
DKFLKLNGEELKKLPAPEVAVNYYMNEDLYMFDEFQTSRAPNSRRPKVDNLYDVFVNVRD
DEAEHCKTMKACQTHETLRSPHAVQSSVEADAE
>TaAOX16
MAASAAPLRAALARSPAPAAARAPPGFLPLPPRARRVRLGPVALVSGRGRRIRAEATART
RQEKEQQEAEVSAVEDSFAVREAAAAPPPEEEGGFDEELTLAGEDGDWVVRFEQSFNVFL
TDTVIFILDILYRDRDYARFFVLETIARVPYFAFISVLHLYETFGWSRRADNIKVHFAES
MNEFHHLLIMEALGGNSVWLDRFLARFSAFFYYFVTVAMYMLSPRMAYHFSECVERHAYS
TYDKFLKLNGEELKKLPAPEVAVNYYMNEDLYMFDEFQTSRAPNSRRPKVDNLYDVFVNV
RDDEAEHCKTMKACQTHETLRSPHAVQSSLEADAE
>TaAOX17
DTVIFILDILYRDRDYARFFVLETIARVPYFAFISVLHLYETFGWSRRADNIKVHFAESM
NEFHHLLIMEALGGNSVWLDRFLARFSAFFYYFVTVGMYMLSPRMAYHFSECVERHAYST
YDKFLKLNGEELKKLPAPEVAVNYYMNEDLYMFDEFQTSRAPNSRRPKVDNLYDVFVNVR
DDEAEHCKTMKACQTHETLRSPHAVQSSIEADAE
>TaAOX18
MAASAAPLRAALARSPAPAAAARTPPAFLPLPPRARGIRLNPVVLASGRGRRIRAEATAR
TRQEKEQQEAEVSAVEDSFPVREAAATPAPEEEGGFDDELTLAGEDGDWVVRFEQSFNIF
LTDTVIFILDILYRDRDYARFFVLETIARVPYFAFISVLHLYETFGWSRRADNIKVHFAE
SMNEFHHLLIMEALGGNSVWLDRFLARFSAFFYYFVTVGMYMLSPRMAYHFSECVERHAY
STYDKFLKLNGEELKKLPAPEVAVNYYMNEDLYMFDEFQTSRAPNSRRPKVDNLYDVFVN
VRDDEAEHCKTMKACQTHETLRSPHAVQSSIEADAE

Figure S1. This study utilized 81 AOX protein sequences from multiple species
