# Supplementary material for: Exploring Evolutionary Pathways and Abiotic Stress Responses through Genome-Wide Identification and Analysis of the Alternative Oxidase (AOX) Gene Family in Common Oat (Avena sativa)
Source: Int J Mol Sci. 2024 Aug 29;25(17):9383. doi: 10.3390/ijms25179383 (PMC11395127; doi:10.3390/ijms25179383)
Supplement: Supplementary file 1 [file ijms-25-09383-s001.zip › Figure S3.pdf]

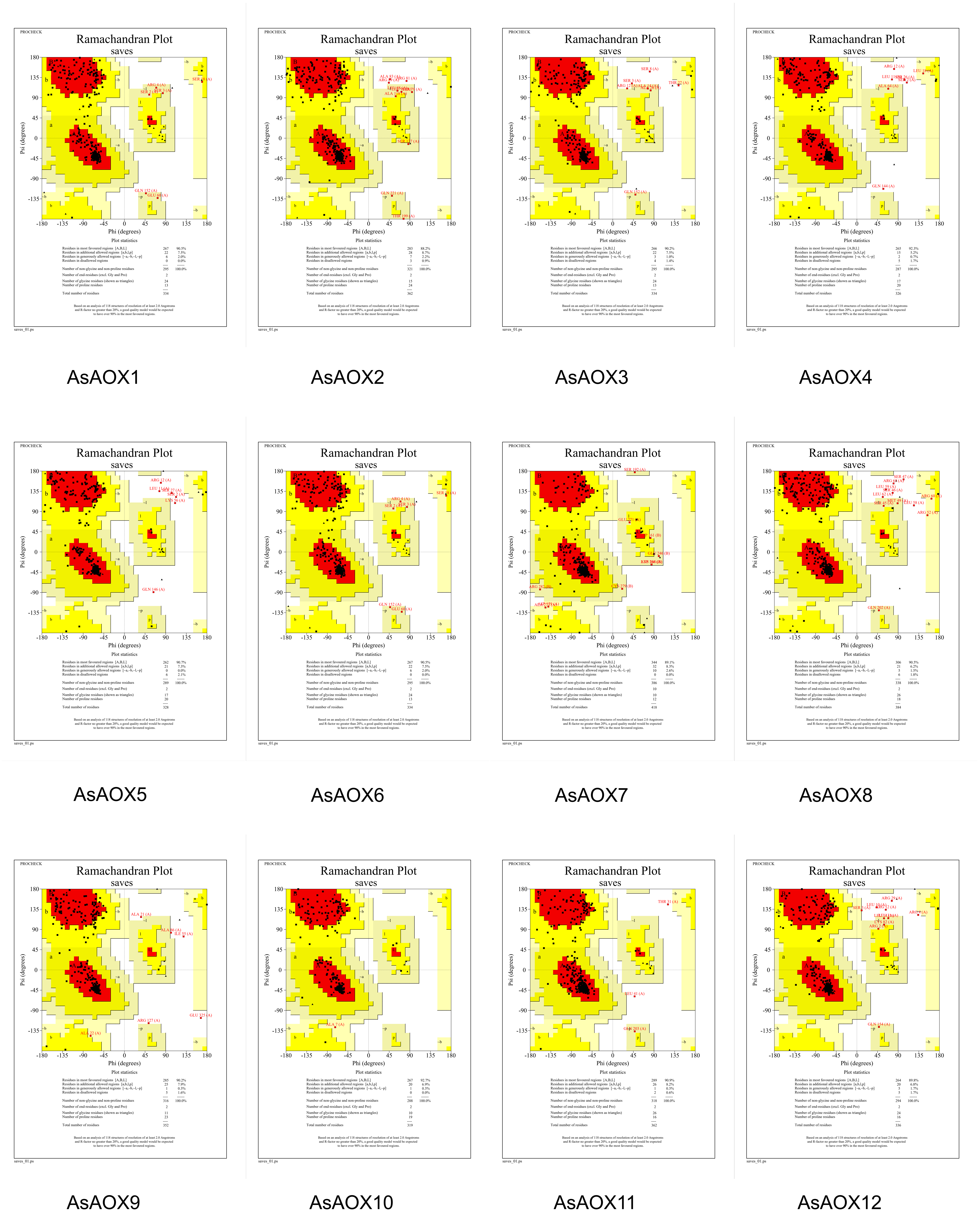

Figure S3. Ramachandran plot assessments of homology models for the 12 AsAOX gene members
