# Supplementary material for: Exploring Evolutionary Pathways and Abiotic Stress Responses through Genome-Wide Identification and Analysis of the Alternative Oxidase (AOX) Gene Family in Common Oat (Avena sativa)
Source: Int J Mol Sci. 2024 Aug 29;25(17):9383. doi: 10.3390/ijms25179383 (PMC11395127; doi:10.3390/ijms25179383)
Supplement: Supplementary file 1 [file ijms-25-09383-s001.zip › Figure S4.pdf]

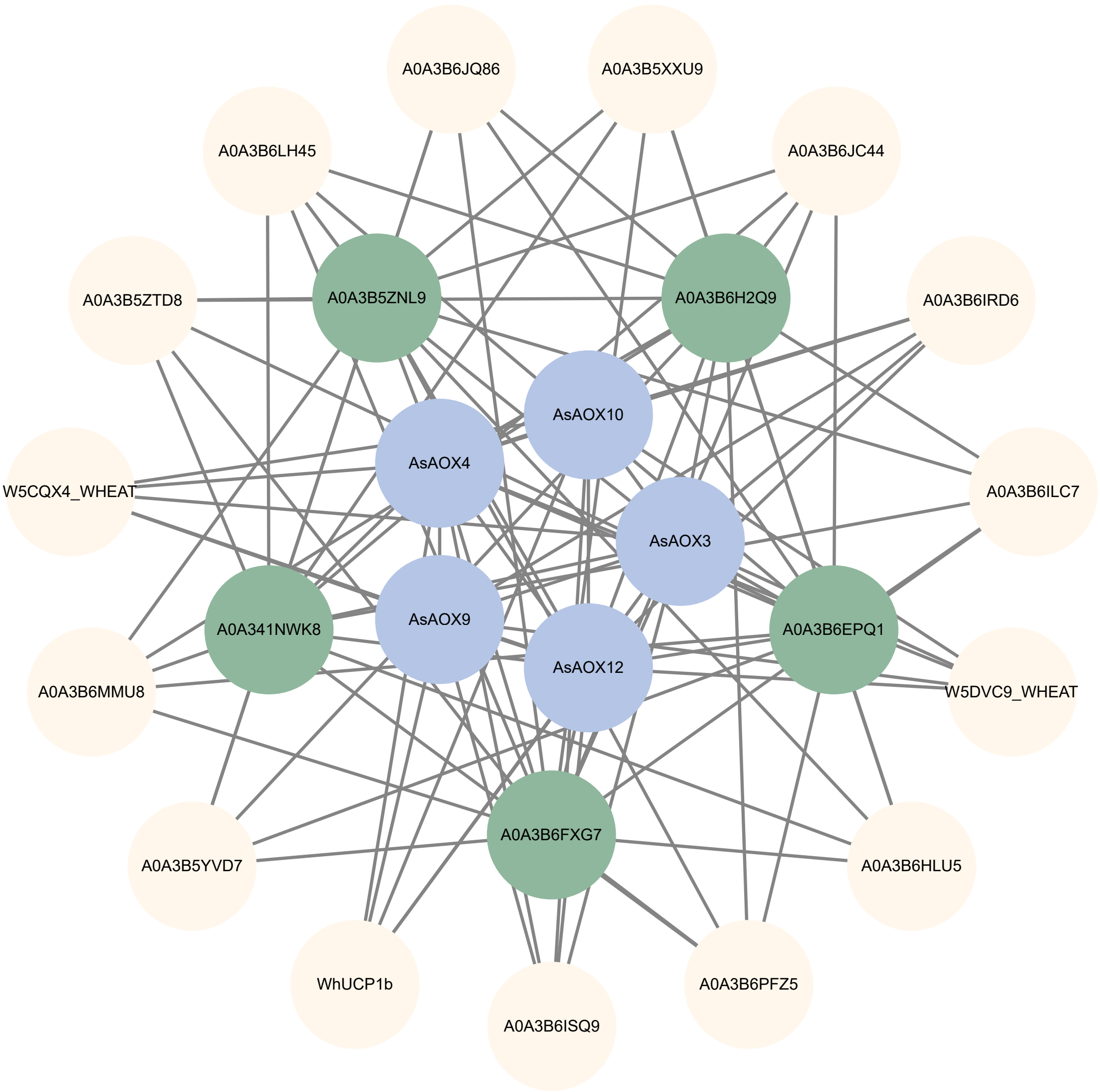

Figure S4. PPI network reveals interaction relationships between AsAOXs proteins and orther proteins. Different colors and positions represent the degree of interaction with AsAOX proteins, with proteins closer to the inner circle showing stronger interactions.
