# Supplementary material for: Exploring Evolutionary Pathways and Abiotic Stress Responses through Genome-Wide Identification and Analysis of the Alternative Oxidase (AOX) Gene Family in Common Oat (Avena sativa)
Source: Int J Mol Sci. 2024 Aug 29;25(17):9383. doi: 10.3390/ijms25179383 (PMC11395127; doi:10.3390/ijms25179383)
Supplement: Supplementary file 1 [file ijms-25-09383-s001.zip › Figure S6.pdf]

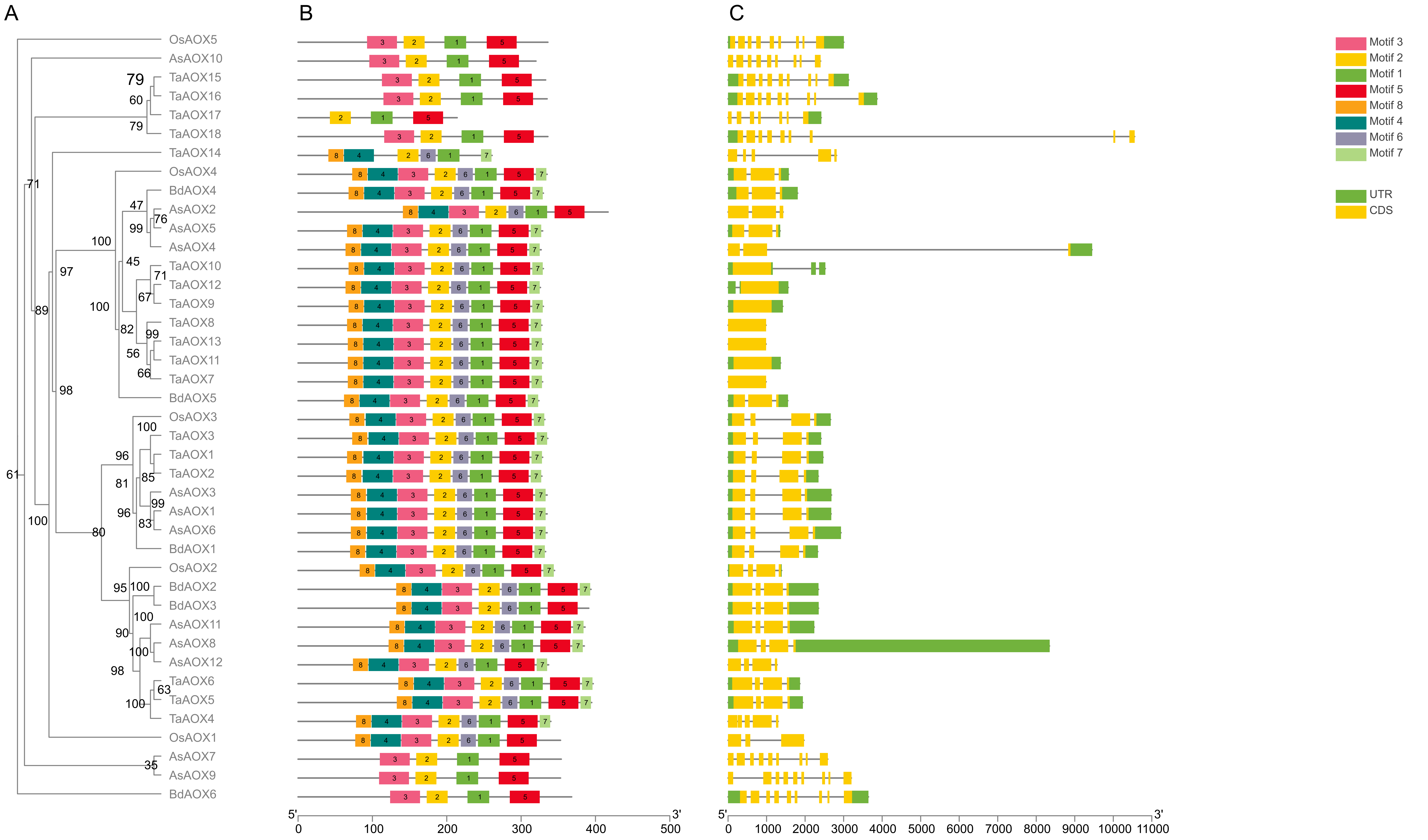

Figure S6 presents the phylogenetic tree (A), conserved motifs (B), and gene structure analysis (C) of 12 AsAOX genes, 5 OsAOX genes, 18 TaAOX genes, and 6 BdAOX genes. Additionally, the numbers on the evolutionary branches represent bootstrap values.
